# Aneuploidy detection in pooled polar bodies using rapid nanopore sequencing

**DOI:** 10.1007/s10815-024-03108-7

**Published:** 2024-04-20

**Authors:** Silvia Madritsch, Vivienne Arnold, Martha Haider, Julia Bosenge, Mateja Pfeifer, Beatrix Weil, Manuela Zechmeister, Markus Hengstschläger, Jürgen Neesen, Franco Laccone

**Affiliations:** 1 Institute of Medical Genetics, Medical University of Vienna, Währinger Straße 10, 1090 Vienna, Austria; 2 HLN-Genetik GmbH, Ortliebgasse 25/1, 1170 Vienna, Austria

**Keywords:** Polar bodies, Nanopore, Aneuploidy, Preimplantation genetic testing for aneuploidy

## Abstract

**Purpose:**

Various screening techniques have been developed for preimplantation genetic testing for aneuploidy (PGT-A) to reduce implantation failure and miscarriages in women undergoing in vitro fertilisation (IVF) treatment. Among these methods, the Oxford nanopore technology (ONT) has already been tested in several tissues. However, no studies have applied ONT to polar bodies, a cellular material that is less restrictively regulated for PGT-A in some countries.

**Methods:**

We performed rapid short nanopore sequencing on pooled first and second polar bodies of 102 oocytes from women undergoing IVF treatment to screen for aneuploidy. An automated analysis pipeline was developed with the expectation of three chromatids per chromosome. The results were compared to those obtained by array-based comparative genomic hybridisation (aCGH).

**Results:**

ONT and aCGH were consistent for 96% (98/102) of sample ploidy classification. Of those samples, 36 were classified as euploid, while 62 were classified as aneuploid. The four discordant samples were assessed as euploid using aCGH but classified as aneuploid using ONT. The concordance of the ploidy classification (euploid, gain, or loss) per chromosome was 92.5% (2169 of 2346 of analysed chromosomes) using aCGH and ONT and increased to 97.7% (2113/2162) without the eight samples assessed as highly complex aneuploid using ONT.

**Conclusion:**

The automated detection of the ploidy classification per chromosome and shorter duplications or deletions depending on the sequencing depth demonstrates an advantage of the ONT method over standard, commercial aCGH methods, which do not consider the presence of three chromatids in pooled polar bodies.

**Supplementary Information:**

The online version contains supplementary material available at 10.1007/s10815-024-03108-7.

## Introduction

Increasing the live birth rate and decreasing the miscarriage rate through in vitro fertilisation (IVF) treatment is a critical focus of reproductive medicine, specifically in combination with advanced maternal age [1]. A study showed that in women of advanced age (between 38 and 41 years), more than three quarters of the embryos produced by IVF were aneuploid, resulting in implantation failure and miscarriage [2]. Many studies indicate that preimplantation genetic testing for aneuploidy (PGT-A) increases pregnancy rates per embryo transfer by analysing the ploidy classification of blastomeres, trophectoderm or polar bodies [3–5]. The first PGT-A was performed by fluorescence in situ hybridisation (FISH), where only a few chromosomes could be evaluated at the same time. Today, array-based methods, e.g. array-based comparative genomic hybridisation (aCGH) or single nucleotide polymorphism (SNP) arrays, and, increasingly, next-generation sequencing (NGS) methods are used [6].

In some countries, the analysis of embryonic cells such as blastomeres or trophectoderm cells is strictly regulated by law. Austrian legislation allows preimplantation genetic testing on trophectoderm only in specific cases outlined in the Reproductive Medicine Act (FMedG). Such indications are three or more IVF procedures where implantation has failed, three or more miscarriages documented, or known chromosomal/genetic abnormality in at least one parent [7]. In the absence of these specified indications, the analysis of polar bodies may represent a possible alternative for PGT-A analysis. In addition, biopsy of polar bodies is already completed 16 h after insemination [8], and thus, fresh embryo transfer is possible [9]. The absence of mosaicism in polar bodies could provide a further advantage, facilitating the decision if the associated embryo should be transferred [10].

Polar bodies develop during oogenesis. Accompanied with the division of homologous chromosomes during meiosis I, the first polar body extrudes, containing two chromatids. After fertilisation, during meiosis II, a second polar body is formed containing a haploid set of chromatids [11]. Several studies showed that analysing both polar bodies using aCGH accurately predicts aneuploidy of maternal meiotic origin in the zygote [12–15]. This method finds support in the European Society of Human Reproduction and Embryology (ESHRE) good practice recommendations for PGT [8, 16]. Notably, this approach does have limitations. A major disadvantage of the analysis of polar bodies is that solely maternal meiotic aneuploidies are identified, neglecting mitotic errors or paternally derived meiotic errors. Furthermore, polar bodies are prone to fragmentation as they begin to degrade after extrusion, potentially affecting the accuracy of the analysis [11, 16]. Some studies have shown discrepancies between the observed copy number in polar bodies and the actual copy number in the embryo. This suggests that factors beyond mitotic errors may be involved. Hypothesised alternative explanations for the discrepancies include aneuploidy in the primary oocyte or chromosome loss during meiosis, such as anaphase lagging [15, 17–19].

The usual method of separately analysing both the first and second polar bodies is costly and complicated, as the results must be combined for a final assessment. It has been shown that analysing pooled polar bodies can reliably predict oocyte ploidy, which can reduce the costs and workload of PGT-A in contrast to separate polar body analysis. However, conventional aCGH analysis software is designed to work with diploid genomes, and it is necessary to adapt the log_2_ ratio thresholds for gains and losses to meet the euploid state in pooled polar bodies with three chromatids [20]. However, the results may be false a positive/negative in case one polar body does not amplify [12].

Over the last few years, WGA-based NGS methods like VeriSeq PGS (Illumina) and Ion SingleSeq™ (Thermo Fisher) sequencing on blastomeres and blastocysts [21–24], oocytes [25] and polar bodies [4] have been increasingly utilised for PGT-A. In contrast to aCGH analysis, NGS methods offer enhanced dynamic resolution [26]. However, established systems are associated with high acquisition costs, and the investment is only worthwhile if a high sample throughput is analysed. In comparison, the portable sequencing device MinION Mk1C from Oxford Nanopore Technologies is more affordable. Nanopore sequencing works by decoding changes of electrical current as a single-stranded DNA or RNA molecule passes through a protein nanopore. It is the only sequencing technology that offers real-time analysis in fully scalable formats with low-cost access [27]. Wei et al. [28] showed in a proof-of-principle study on nine trophectoderm samples that low-pass nanopore sequencing can predict aneuploidy, and very recently, Tan et al. [29] described that ONT had a 97.92% concordance in aneuploidy rates and segmental aneuploidy of 96 trophectoderm cells with the Illumina VeriSeq NGS platform.

For the first time, we present a workflow for PGT-A on polar bodies using rapid nanopore single-cell low-pass whole-genome sequencing. The results are compared with the already published method on pooled polar bodies using aCGH [20]. In addition, it can be shown that structural imbalances can be detected using the nanopore workflow.

## Materials and methods

**Fig. 1:**
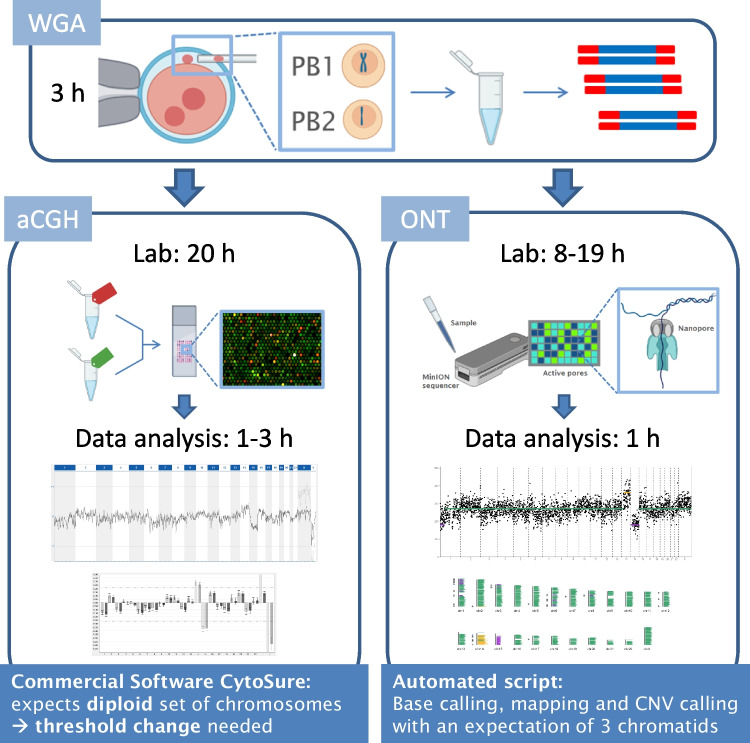
Schematic workflow of the two methods used in this study: array-based comparative genomic hybridisation (aCGH) and the Oxford nanopore technology (ONT). The initial step of both techniques is the biopsy of polar bodies and whole-genome amplification (WGA), followed by method-specific library preparation and analysis of the ploidy classification and copy number variations (CNVs)

A schematic workflow of both methods that were compared in this study, aCGH and nanopore, is shown in Fig. 1. The biopsy of polar bodies and whole-genome amplification (WGA) are the initial steps of both techniques, followed by method-specific library preparation and analysis.

### Patients and sample collection

In this study, the first and second polar bodies of 108 oocytes were biopsied and transferred together into a micro-tube containing 2.5 µl of phosphate-buffered saline (PBS). From those, six euploid samples were used to normalise the ONT data and excluded from analysis. During the study period, on average, 4 samples per patient were tested at the HLN-Genetik GmbH. The average age of the patients per sample was 40.2 years. The samples were randomly selected from a cohort in which all patients provided informed consent. The DNA of the pooled polar bodies was then amplified using a PicoPLEX WGA Kit (Takara, #R30050) following the manufacturer’s protocol. As a quality control, 2 µl of the amplified DNA (WGA-DNA) was separated by gel electrophoresis. Furthermore, the yield of the amplified DNA was measured with a Qubit 2.0 fluorometer using a Qubit dsDNA High Sensitivity Assay Kit (Thermo Fisher, Q32851). The amplified DNA was then used for both aCGH and nanopore analysis (Fig. 1).

### Array-based comparative genomic hybridisation (aCGH)

For aCGH, whole-genome-amplified male/female control DNA and sample DNA were labelled with a CytoSure Genomic DNA Labelling Kit (Oxford Gene Technology, Part No. 500040) and hybridised to a DNA microarray (Oxford Gene Technology, CytoSure Embryo Screen Array) for 16 h following the manufacturer’s protocol. The intensity of fluorescence signals was measured by scanning the microarray slides with a DNA microarray scanner (Innopsys, InnoScan 710), followed by feature extraction with Mapix software (Innopsys, version 8.5.0). The quantified features were then analysed and interpreted with CytoSure Interpret software (Oxford Gene Technology, version 4.10.44).

For a diploid set of chromosomes, the mean log_2_ ratio (sample vs. control DNA amplified by WGA) for a gain is expected to be 3:2, with log_2_(3/2) = 0.58 and for a loss 1:2, with log_2_(1/2) =  − 1. The expected mean log_2_ ratio values for a gain for polar bodies that contain three chromatids would be log_2_(4/3) = 0.42 and for a loss log_2_(2/3) =  − 0.58. Consequently, we defined the threshold for a gain as the expected boundary between euploid and gain corresponding to log_2_(3.5/3) = 0.22 and between euploid and loss to log_2_(2.5/3) =  − 0.26. An autosome is assessed as aneuploid, if the mean log_2_ ratio sample vs. controls (female and male) is above or below the defined thresholds. For the evaluation of chromosome X only, the mean log_2_ ratio sample vs. female control is considered. The aCGH results are further clinically evaluated by a trained personal.

### Nanopore (ONT)

The amplicon fragment lengths were determined using TapeStation gDNA Screen Tape (Agilent). To eliminate fragments under 150 bp, 30 µl of WGA-DNA underwent left-side size selection with 1.2-fold SPRIselect reagent (Beckman Coulter, #REFB23318). The size-selected DNA was eluted in 25 µl Buffer EB (Qiagen, #19,086).

#### DNA repair and end-prep, native barcoding and adapter ligation

For the “native barcoding genomic DNA” workflow (Oxford Nanopore, SQK-LSK109), 80 ng of size-selected WGA-DNA was processed with DNA repair and end-prep with the “NEBNext Companion Module” (NEB, #E7180S) and purified with onefold AMPure XP Beads (Beckman Coulter, #A63881). Afterwards, native barcode (Oxford Nanopore, #EXP-NBD104, #EXP-NBD114) and NEB Blunt/TA Ligase Master Mix (NEB, #M0367L) were added, and the purification step was repeated.

For adapter ligation with six equimolar pooled barcoded samples, Adapter Mix II (Oxford Nanopore, SQK-LSK109) and (5 ×) NEBNext Quick Ligation Rxn Buffer (NEB, #B6058S) were incubated and purified using 0.5-fold AMPure XP beads, followed by elution in Buffer EB (Oxford Nanopore, SQK-LSK109).

#### MinION sequencing

For sequencing on a MinION MK1C (Oxford Nanopore), MinION Flow Cells (R9.4.1 flow cells with at least 1300 pores found during flow cell check) were loaded with priming mix (Oxford Nanopore, EXP-FLP002), followed by sequencing of the library mix containing the DNA library (~ 160 ng) from the previous step, sequencing buffer and loading beads for 16 h at − 180 mV.

#### Data analysis

Fast5 files were basecalled using guppy_basecaller (version 4.2.2) [30], concatenated and then aligned to the GRCh38 assembly using minimap2 (version 2.17) [30]. The SAM files were converted to BAM format and sorted using SAMtools (version 1.10) [31], including removal of reads with a mapping quality score below 5.

For the computation of the binned read count data and copy number variation (CNV) states, an adapted version of the function *Aneufinder* from the R package AneuFinder (version 1.22.0) [32, 33] was used. For mappability correction and blacklisting, six euploid samples with a derivative log_2_ ratio (DLR) spread below 0.7 were distinguished using the above-described aCGH method, and the associated BAM files were merged using SAMtools. This reference file was then used for blacklisting. Reads were counted in fixed-width windows (bins) of 100 kb, and bins with a read count above the 0.9985 or below the 0.1 quantile were excluded [32]. Mappability correction via a variable-width binning approach was applied using the abovementioned reference BAM file. The bin size was set to 1 Mb, and GC correction was performed. For the computation of CNV states and breakpoints, a hidden Markov model with possible states ranging from 0- to 4-somy and zero-inflation was used. The parameter *most.frequent.state* was adjusted to 3-somy to match the expected sum of three chromatids for a euploid sample. CNV segments were filtered for a minimal segment size of 10 Mb. All other parameters were left at the default settings. Reference samples were excluded from aneuploidy analysis. The aneuploidy per chromosome is computed with the function *heatmapAneuploidies* from the R package AneuFinder that computes the most frequent state for each chromosome. For the single-chromosome view, the R package karyoploteR (version 1.28.0) was used [34]. Additionally, the basecalled and concatenated FASTQ files were randomly downsampled to 300 k reads using seqtk (version 1.3-r106) [35]; the above-described analysis was repeated with the reduced data set.

## Results

We tested the possibility and reliability of nanopore sequencing as an alternative to the current aCGH method for PGT-A on 102 pooled polar bodies.

The DLR spread, which measures the spread of the difference in log_2_ ratios between adjacent binding spots along the genome, is an important quality metric for aCGH data. Lower DLR spread values allow higher resolution analysis of CNVs. The mean DLR spread of all 102 samples was 0.57 ± 0.10 (mean ± SD). The values were comparable to the average quality values of pooled polar bodies based on aCGH analyses performed in our laboratory. While a DLR spread for genomic DNA above 0.3 is considered poor, for single cells, much higher DLR spreads (0.64–1.84) are observed [36]. With the ONT workflow, 1.08 ± 0.42 Mb (mean ± SD) reads per sample with an average read length ranging from 520 to 590 bp could be sequenced. On average, 89% of the sequenced reads could be mapped and utilised for CNV detection with AneuFinder (Supplementary Table S1).

### Aneuploidy detection—aCGH vs. ONT

**Fig. 2:**
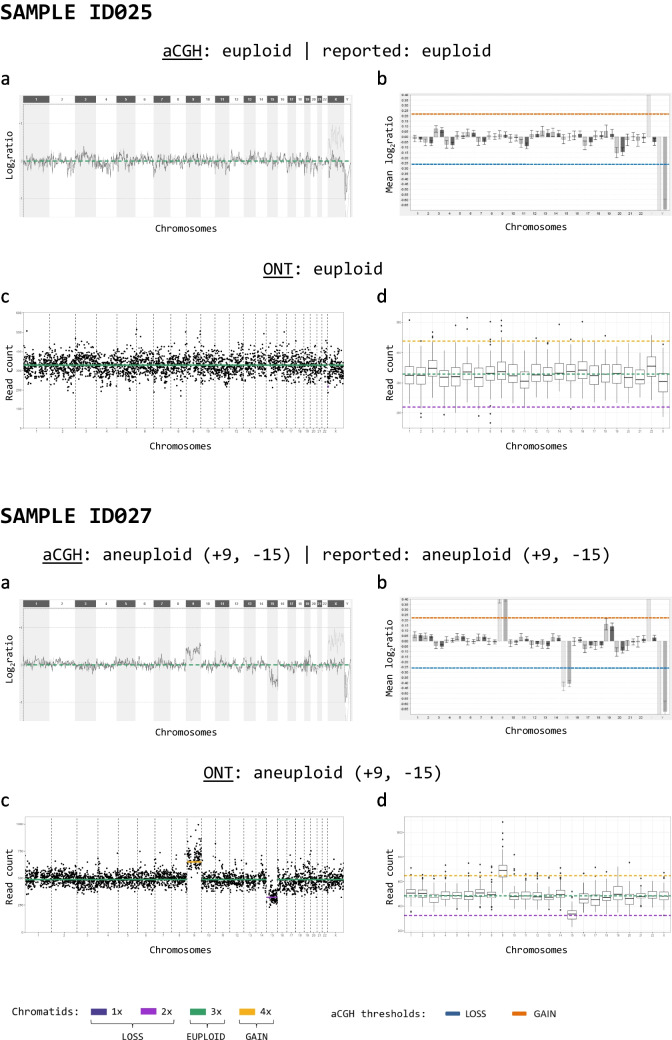
Comparing ploidy analysis using aCGH and ONT for two selected samples. The ploidy classification based on the mean log_2_ ratio thresholds for aCGH and the reported clinical evaluation or the computed ploidy classifications with ONT are stated above. (a) Genome-wide analysis of the CNV state using aCGH. (b) Mean log_2_ ratios (sample vs. control DNA amplified by WGA) of each chromosome using aCGH. Light grey bars show signal distribution vs. male control and dark grey bars that vs. female control. Thresholds for a gain (orange line) and loss (blue line) for pooled polar bodies are indicated. (c) Genome-wide CNV analysis using ONT. Black dots represent the total read count per variable-width bin of approximately 1 Mb. The yellow line indicates a gain, and the violet line indicates a loss, respectively. (d) Boxplot of binned read counts per chromosome using ONT. Coloured lines show the expected read count per state. The yellow line indicates the expected read count for a gain, and the violet line indicates the expected read count for a loss, respectively

The signal pattern (binding spot signal log_2_ ratio in aCGH and read count per bin in ONT) over the whole genome was visually comparable, as was the mean value of each chromosome (Fig. 2). In 96% (98/102) of all analysed samples, the ploidy classification (euploid, aneuploid) was in concordance between ONT and aCGH. Of those in concordance, 36 samples were classified as euploid, while 62 samples were classified as aneuploid (Table 1 and Supplementary Table S1). The four discordant samples (ID023, ID028, ID062, ID072) were all classified as euploid using aCGH and aneuploid using ONT (Supplementary Fig. S1). Clinical evaluation showed that only two samples, ID023 and ID062, are evaluated differently to ONT, and two others (ID006 and ID063) are clinically evaluated as unsure (Supplementary Table S1).

**Table 1:**
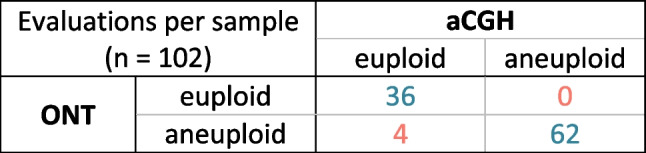
Number of samples assigned to ploidy classifications for the two methods in the form of a cross table (blue, concordant; red, discordant)

### Aneuploidy statistics per chromosome

The concordance of the ploidy state classification (euploid, gain, loss) for each chromosome was assessed. In 92.5% (2169 of 2346) of analysed chromosomes, the ploidy classification per chromosome was concordant between aCGH and ONT. With both methods, the classification of 2032 chromosomes was assessed as euploid, a chromatid gain was identified 47 times, and at least one chromatid loss was identified 90 times. A total of 94.0% (47/50) of chromatid gains, 93.7% (2032/2168) of euploid evaluations and 70.3% (90/128) of chromatid losses classified via aCGH were in concordance with those classified using ONT (Table 2).

**Table 2:**
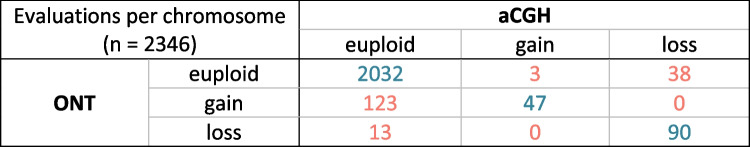
Number of chromosomes assigned to specific ploidy classifications for both methods in the form of a cross table (blue, concordant; red, discordant)

**Fig. 3:**
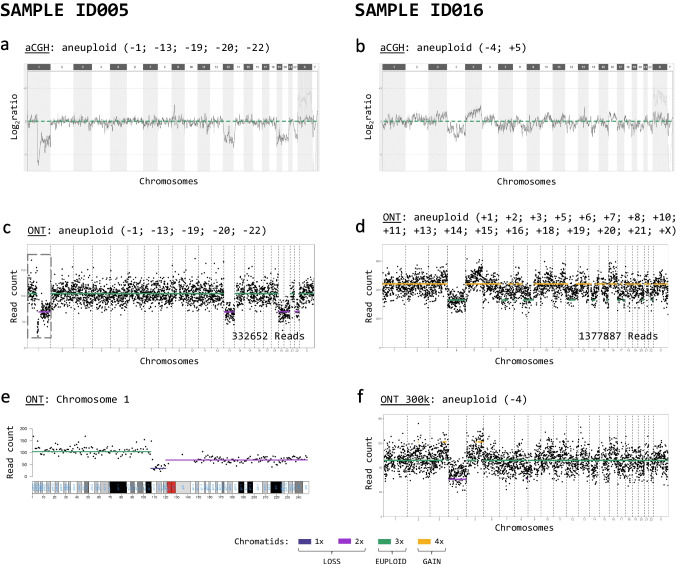
Segmental aneuploidy detection (ID005) and baseline shift (ID016). For **a**–**d** and **f**, the computed ploidy classifications with ONT or the ploidy classification based on the mean log_2_ ratio thresholds for aCGH, which are consistent with the reported clinical evaluation, are stated above. **a**, **b** Genome-wide analysis of the CNV state using aCGH for the two selected samples. **c**, **d** Genome-wide CNV analysis using ONT. Black dots represent the total read count per variable-width bin of approximately 1 Mb. The yellow line indicates a gain, and the violet line indicates a loss or double loss, respectively. **e** Single chromosome view of the CNV analysis from above (sample ID005) showing the partial loss in chromosome 1. **f** Genome-wide CNV analysis using ONT with the data set of sample ID016 reduced to 300 k reads

**Fig. 4:**
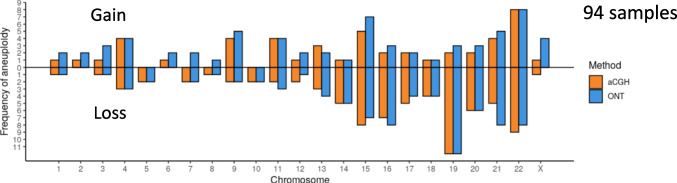
Absolute numbers of chromosomal gains and losses per method used, including 94 samples (highly complex samples were filtered)

There were few samples with a high rate of discrepant chromosomal ploidy classifications. We observed in most of those samples that the baseline of the ONT data was at a different level than that of the aCGH data. For example, for sample ID016, shifting of the ONT baseline according to that of aCGH reduced the discrepancy from 17 to only 1 chromosome (Fig. 3b, d, f). Therefore, we excluded eight samples with more than half of the chromosomes assigned as aneuploid using ONT for further concordance analysis. Despite this discrepancy, all eight samples were initially evaluated as aneuploid with both methods. The exclusion affected mainly samples with a visually shifted baseline in the ONT results towards lower read counts, increasing the number of assigned chromosomal gains extensively (Supplementary Fig. S2). The concordance per chromosome increased to 97.7% (2113/2162) (Table 3) without the eight samples. Specifically, the number of chromosomes assigned as euploid using aCGH but as gain using ONT was reduced to 21 (previously 123), and the number of chromosomes assigned as loss in aCGH but euploid using ONT was reduced to 12 (previously 38) (Table 2 vs. Table 3). A detailed evaluation per chromosome and per sample is shown in Supplementary Table S1. In general, the absolute number of chromosomes assigned as aneuploid was higher using ONT than using aCGH (150 vs. 131). The chromosomes with the highest rates of aneuploidy assignment were 15, 16, 19, 21 and 22 in both methods. The total number of assigned chromatid losses (ONT: 84, aCGH: 83) was higher than chromatid gains (ONT: 66, aCGH: 48) in both methods (Fig. 4).

**Table 3:**
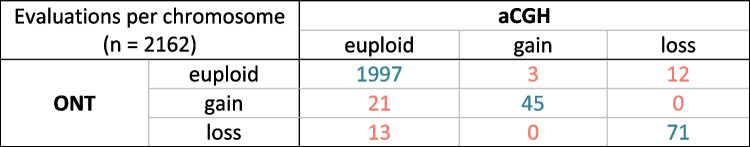
Number of chromosomes assigned to specific ploidy classifications for both methods in the form of a cross table after filtering highly complex samples (blue, concordant; red, discordant)

### Segmental gains and losses

The ONT workflow can automatically identify small aberrations, such as a gain of 50 Mb in chromosome 2 of sample ID050, a loss of approximately 13 Mb in chromosome 1 of sample ID005 and a gain of 2p, a loss of 7p and a gain of Xq in sample ID072. These aberrations could be seen in the aCGH data by visually reviewing the genome-wide analysis results (Fig. 3a, c, e and Supplementary Fig. S3a-c).

### Required number of reads and workflow times

The aCGH workflow takes approximately 24 h, including an overnight hybridisation step of 16 h. The ONT workflow can sequence and analyse approximately 1 M reads in a similar time frame. As the workflow time is important if a fresh embryo transfer is planned, we checked whether we can reduce the number of reads and thus decrease the sequencing time. To simulate a shorter sequencing time, the number of reads was randomly downsampled to 300 k, achieving a sequencing time of approximately 5 h (Fig. 1). After analysing the reduced data set, the per-sample ploidy classifications relative to the original ONT results were equal, except for two samples where the loss in chromosome 21 was less than half of the length of the chromosome and thus not predicted as loss, while in the other sample, the ploidy classification was assigned as aneuploid using the reduced data set, unlike in the original result and the aCGH evaluation (Supplementary Fig. S4). Per-chromosome ploidy classifications were concordant in 94.4% (2215/2346) of chromosomes with respect to the original ONT analysis and in 94.7% (2222/2346) with respect to the aCGH results (Supplementary Table S2a and b). Segmental aneuploidy in samples ID005, ID050 and ID072 could also be detected with the reduced data sets (Supplementary Fig. S3d and e). Additionally, for a few samples, such as ID016, the baseline was now set at the expected read count level (Fig. 3f vs. d).

## Discussion

Here, we show for the first time that aneuploidy detection in pooled first and second polar bodies is possible and reliable using rapid nanopore low-pass sequencing. The distribution of reads is highly similar to the aCGH signals, and the overall ploidy statistics of ONT reached up to 97.7% concordance with the already published aCGH method [20], with a higher rate for aneuploidy assignment using ONT. Our study shows an overall euploidy rate of approximately 35%, with an expected patient mean age per sample of 40 years. According to Demko et al. [37], women aged 38 to 40 have a 40% proportion of euploid embryos, which aligns with our euploidy rates. The aneuploidy rate is the highest for chromosomes 15, 16, 19, 21 and 22, which has been also observed in other studies, where those five chromosomes showed the highest rates of aneuploidy in blastomere and trophectoderm cells [38, 39]. We further found a higher frequency of chromatid losses over gains, which would further lead to an overrepresentation of trisomies than monosomies in the embryos. Reasons for this could be a monosomy in the primary oocyte or a loss of chromatids during meiosis, both leading to absent chromatids in the first polar body even though the oocyte is euploid, illustrated in Fig. 4 in Verdyck et al. [15].

Most challenges encountered in this study are independent of the two methods used. While using polar bodies for PGT-A precludes the difficult interpretation of mosaicism [10], the DNA amplification at the single-cell level is the major critical step and results in much more signal scattering compared to other analyses of multicellular samples, such as blastomeres and trophectoderm, or genomic DNA samples [36]. Additionally, polar bodies start degrading as soon as they are extruded [16] which can lead to further uneven amplification and subsequently to possible borderline results (probe-signal log_2_ ratio or read counts between the two states). To address these issues in clinical settings, trained personnel visually evaluate all data. If there are doubts about the ploidy classification, a statement is added to the final report. For instance, the ploidy classification of chromosome 5 in sample ID028 and subsequently the overall ploidy classification remains unclear due to the undulating patterns (signal intensity around the border of two states) in both aCGH and ONT data sets.

Since these issues, degradation of polar bodies and uneven amplification at single-cell level, make correct and distinct ploidy classification challenging, they may be also responsible for some of the discordant results between aCGH and ONT (e.g. sample ID023 and ID028 in Supplementary Fig. S1). Another reason for the discrepancy between aCGH and ONT analysis could be the baseline shift that occurs when using ONT. In aCGH, the baseline is defined by an equalisation test to control the fluorescence intensity signal [40], while ONT with the hidden Markov model for CNV calling uses the most frequent state parameter to converge to the best fit and attempt to set the baseline at the correct place [32]. In most cases, a baseline shift does not influence the overall ploidy classification (Supplementary Fig. S2). A third reason for the disagreement might be the fixed threshold based on the mean log_2_ ratios of one chromosome of the aCGH data [20], where chromosomes with a partial deletion or gain most likely are not above the threshold. An example is sample ID072, where the aCGH algorithm classified the sample as euploid but clinical interpretation and the ONT CNV caller clearly identifies a gain of 2p, a loss of 7p and a gain of Xq (Supplementary Fig. S1). Still, the patterns of probe-signal log_2_ ratio and read counts are highly similar in all samples.

Besides the principal challenges, the ONT workflow offers a dynamic resolution adjustment. By increasing the sequencing time and reducing the bin size, smaller CNVs may be identified, while the resolution of aCGH is defined by the density of binding spots on the microarray slide. The ONT software can automatically create a detailed sample report optimised for the presence of three chromatids in pooled polar bodies. Figures such as whole genome profiles, boxplots with the read counts per chromosome and views of flagged single chromosomes can be arranged. Furthermore, if the CNV algorithm does not provide a correct representation of the ploidy classifications (e.g. baseline shift, visually wrong CNV calls), the bin size or segment merging size can be changed easily, which might help to improve the ONT results. If the polar bodies are analysed separately, the ONT workflow can be easily adjusted by changing the parameter with the expected number of chromatids. An additional benefit of the ONT workflow is the multifunctionality of the MinION MK1C and the lower purchase cost (currently around 5000 € with six flow cells included) compared to a two-colour microarray scanner (around 90,000 €) as well as its maintenance costs and other equipment required for the aCGH workflow.

To shorten the turn-around time, we were able to show that 300 k reads are sufficient to detect CNVs down to at least 50 Mb. The ONT workflow can sequence and analyse 300 k reads in approximately 12 h, which is approximately half the time of a standard aCGH workflow. Furthermore, library preparation (DNA end-prep, native barcoding, adapter ligation, etc.) can be automated using a pipetting robot, which decreases the hands-on time and potentially reduces possible hands-on variability with respect to aCGH analysis. Since both methods are based on the same WGA system, one method can be used as a backup system for the other.

It is important to note that this study is not without limitations. In general, PGT-A using polar bodies cannot detect paternally derived aneuploidy nor mitotic errors, unlike the more common PGT-A on trophectoderm or blastomeres. Specifically, by analysing pooled polar bodies, there is the possibility that only one polar body is tubed initially or the amplification of one polar body fails completely. Both cases would not be detected using either ONT or aCGH analysis and might lead to false ploidy classification. Furthermore, an additional set of chromosomes (e.g. triploidy) cannot reliably be detected using either aCGH or read counting-based sequencing methods as no additional SNP-based analysis is performed [41]. Discordance between ploidy classifications (euploid/aneuploid) is mainly based on borderline results, undulating patterns and/or signal scattering. A main limitation of this study is that we cannot validate the results by analysis of embryonic material, primarily due to legal restrictions. Consequently, discordant evaluation between aCGH and ONT shown in Supplementary Fig. S1 will remain unresolved. Furthermore, it is not possible to study the subsequent live birth rates, as the samples have been anonymised.

This work is a proof-of-principle study and shows comparable ploidy and CNV classifications using the PicoPlex WGA kit, MinION sequencing for 16 h and CNV analysis using the R package AneuFinder compared to aCGH analysis. Further investigations need to be performed to validate the ploidy classifications in the embryo as well as to define quality thresholds and the resolution limit of this method.

### Supplementary Information

Below is the link to the electronic supplementary material.

Supplementary file1 (PPTX 1.56 MB)

Supplementary file2 (PPTX 1.21 MB)

Supplementary file3 (PPTX 1.51 MB)

Supplementary file4 (PPTX 960 KB)

Supplementary file5 (XLSX 22.4 KB)

Supplementary file6 (XLSX 10.7 KB)

Supplementary file7 (DOCX 15.0 KB)

## Data Availability

The data that support the findings of this study are available upon reasonable request.
