# Supplementary material for: Aneuploidy detection in pooled polar bodies using rapid nanopore sequencing
Source: J Assist Reprod Genet. 2024 Apr 20;41(5):1261–71. doi: 10.1007/s10815-024-03108-7 (PMC11143085; doi:10.1007/s10815-024-03108-7)
Supplement: Supplementary file 1 — Supplementary file1 (PPTX 1.56 MB) [file 10815_2024_3108_MOESM1_ESM.pptx]

## Slide 1
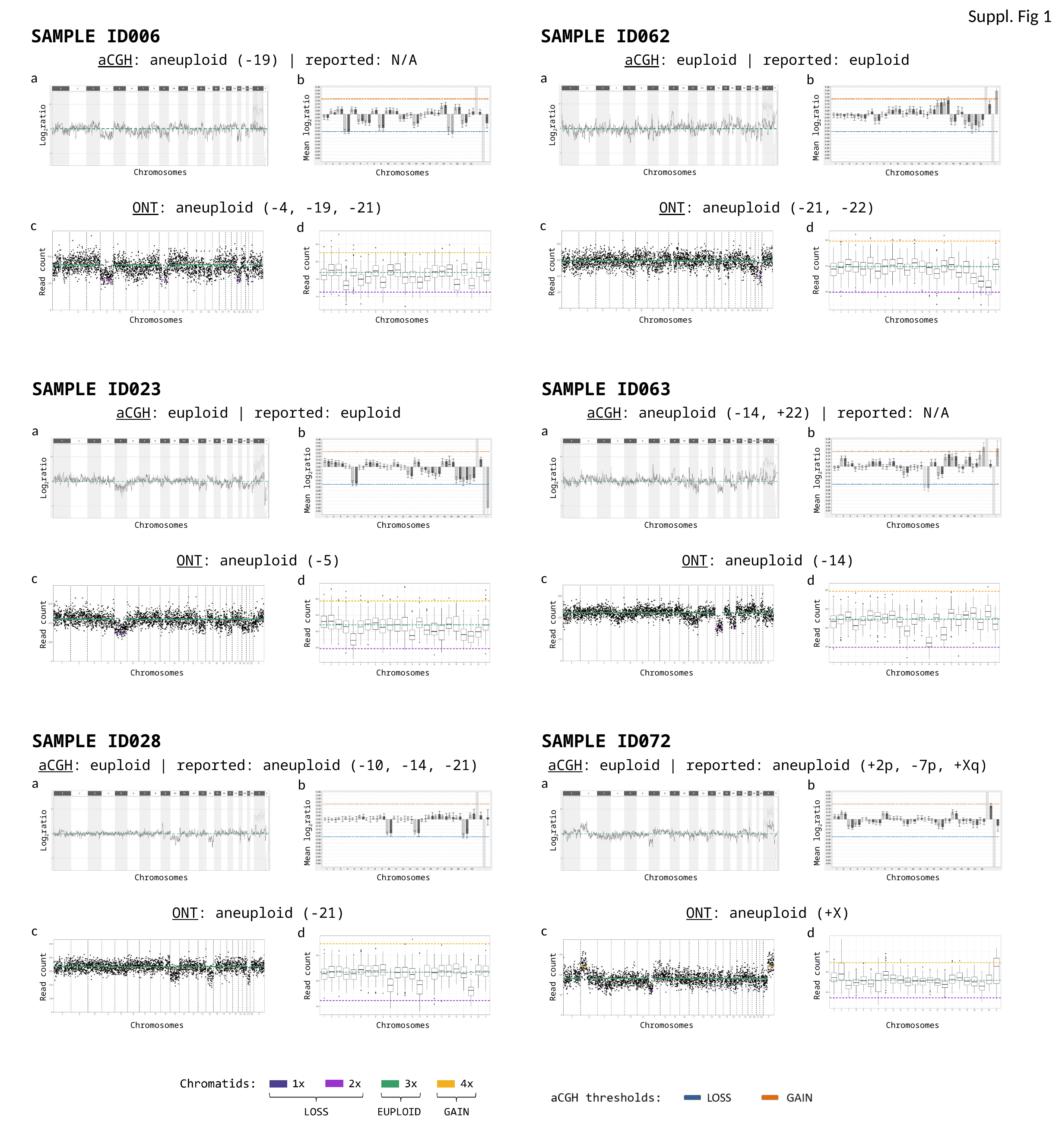

Suppl. Fig 1
SAMPLE ID006
aCGH: aneuploid (-19) | reported: N/A
a
b
Log2ratio
Mean log2ratio
Chromosomes
Chromosomes
ONT: aneuploid (-4, -19, -21)
c
d
Read count
Read count
Chromosomes
Chromosomes
SAMPLE ID062
aCGH: euploid | reported: euploid
a
b
Log2ratio
Mean log2ratio
Chromosomes
Chromosomes
ONT: aneuploid (-21, -22)
c
d
Read count
Read count
Chromosomes
Chromosomes
SAMPLE ID023
aCGH: euploid | reported: euploid
a
b
Log2ratio
Mean log2ratio
Chromosomes
Chromosomes
ONT: aneuploid (-5)
c
d
Read count
Read count
Chromosomes
Chromosomes
SAMPLE ID063
aCGH: aneuploid (-14, +22) | reported: N/A
a
b
Log2ratio
Mean log2ratio
Chromosomes
Chromosomes
ONT: aneuploid (-14)
c
d
Read count
Read count
Chromosomes
Chromosomes
SAMPLE ID028
aCGH: euploid | reported: aneuploid (-10, -14, -21)
a
b
Log2ratio
Mean log2ratio
Chromosomes
Chromosomes
ONT: aneuploid (-21)
c
d
Read count
Read count
Chromosomes
Chromosomes
SAMPLE ID072
aCGH: euploid | reported: aneuploid (+2p, -7p, +Xq)
a
b
Log2ratio
Mean log2ratio
Chromosomes
Chromosomes
ONT: aneuploid (+X)
c
d
Read count
Read count
Chromosomes
Chromosomes
