# Supplementary material for: Aneuploidy detection in pooled polar bodies using rapid nanopore sequencing
Source: J Assist Reprod Genet. 2024 Apr 20;41(5):1261–71. doi: 10.1007/s10815-024-03108-7 (PMC11143085; doi:10.1007/s10815-024-03108-7)
Supplement: Supplementary file 2 — Supplementary file2 (PPTX 1.21 MB) [file 10815_2024_3108_MOESM2_ESM.pptx]

## Slide 1
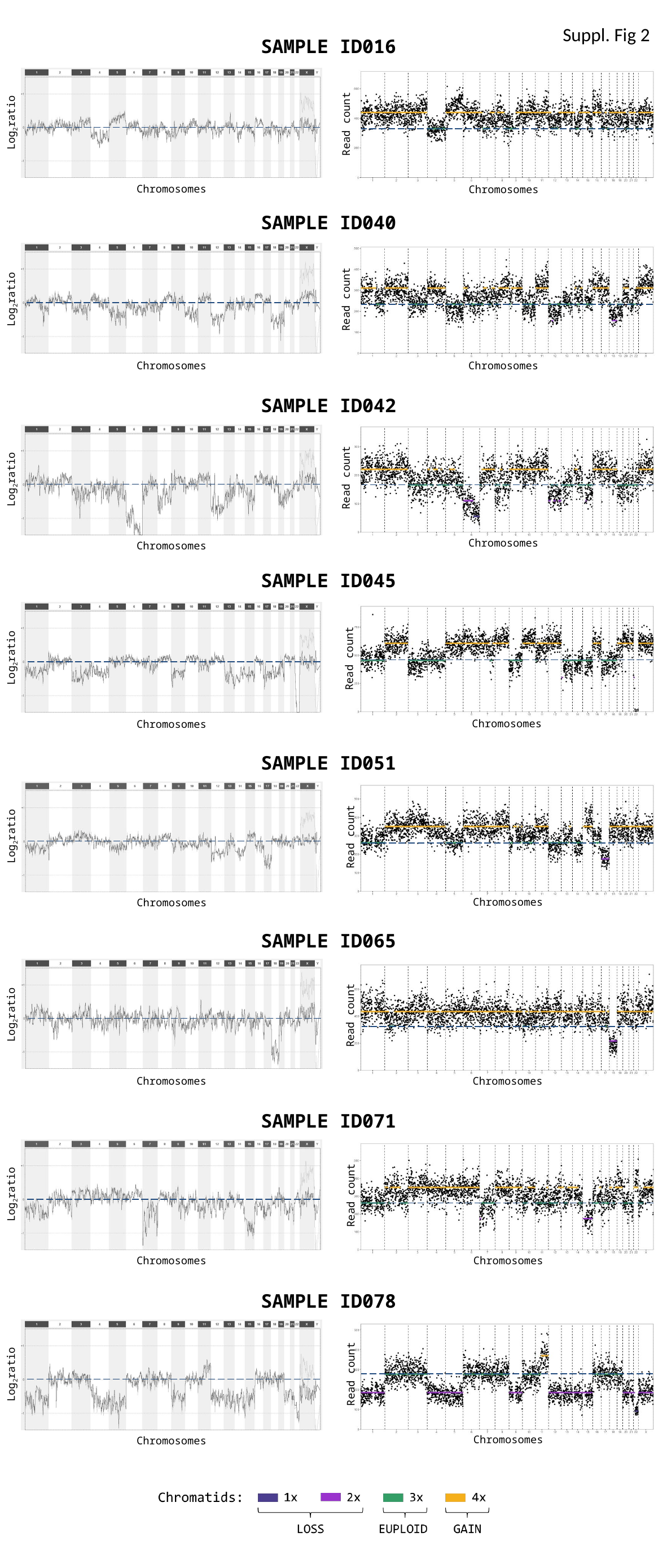

Suppl. Fig 2
SAMPLE ID016
Log2ratio
Read count
Chromosomes
Chromosomes
SAMPLE ID040
Read count
Log2ratio
Chromosomes
Chromosomes
SAMPLE ID042
Read count
Log2ratio
Chromosomes
Chromosomes
SAMPLE ID045
Log2ratio
Read count
Chromosomes
Chromosomes
SAMPLE ID051
Log2ratio
Read count
Chromosomes
Chromosomes
SAMPLE ID065
Log2ratio
Read count
Chromosomes
Chromosomes
SAMPLE ID071
Log2ratio
Read count
Chromosomes
Chromosomes
SAMPLE ID078
Read count
Log2ratio
Chromosomes
Chromosomes
