# Supplementary material for: Aneuploidy detection in pooled polar bodies using rapid nanopore sequencing
Source: J Assist Reprod Genet. 2024 Apr 20;41(5):1261–71. doi: 10.1007/s10815-024-03108-7 (PMC11143085; doi:10.1007/s10815-024-03108-7)
Supplement: Supplementary file 3 — Supplementary file3 (PPTX 1.51 MB) [file 10815_2024_3108_MOESM3_ESM.pptx]

## Slide 1
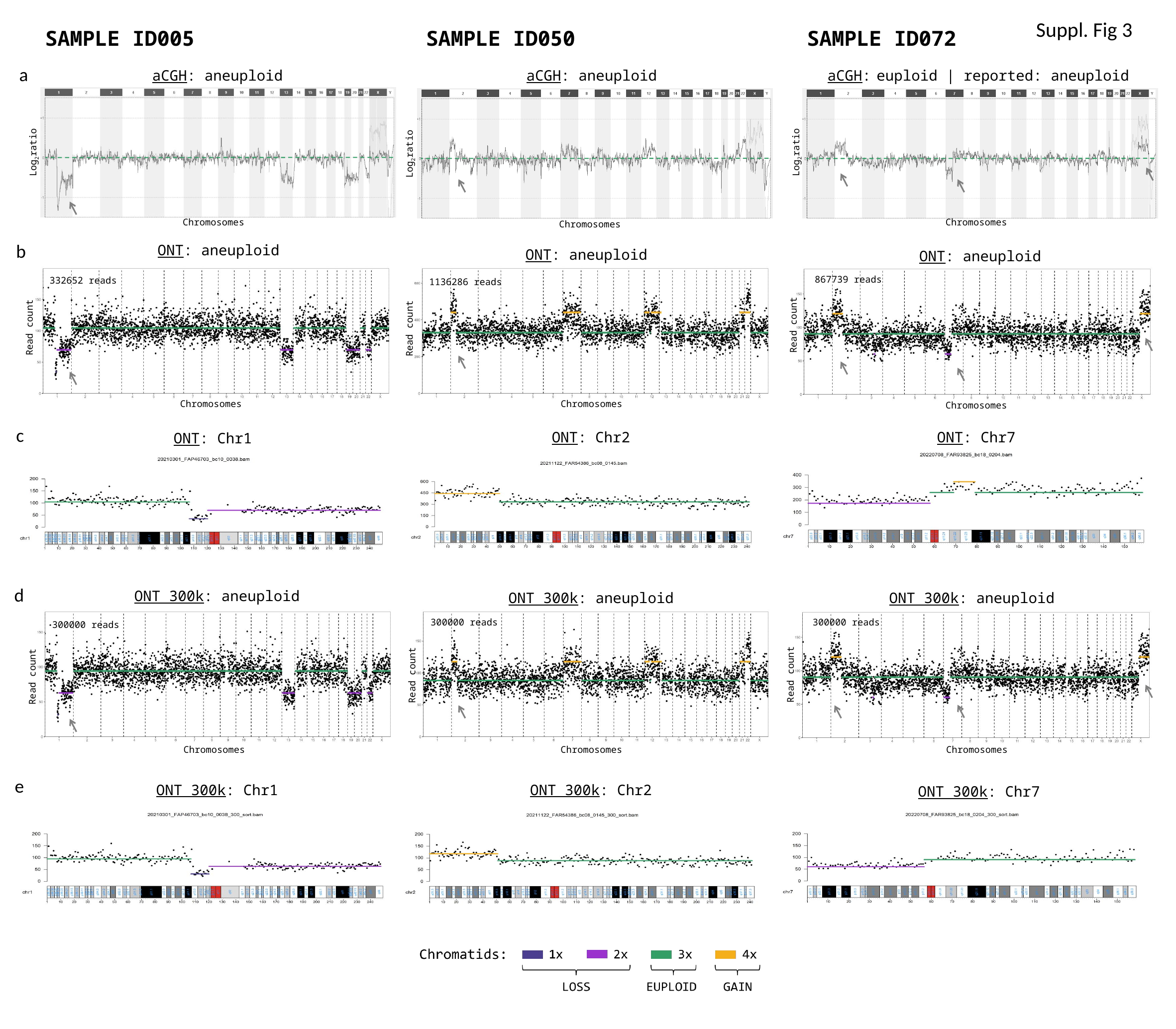

Suppl. Fig 3
SAMPLE ID005
SAMPLE ID050
SAMPLE ID072
a
aCGH: aneuploid
aCGH: euploid | reported: aneuploid
aCGH: aneuploid
Log2ratio
Log2ratio
Log2ratio
Chromosomes
Chromosomes
Chromosomes
b
ONT: aneuploid
ONT: aneuploid
ONT: aneuploid
867739 reads
332652 reads
1136286 reads
Read count
Read count
Read count
Chromosomes
Chromosomes
Chromosomes
c
ONT: Chr2
ONT: Chr7
ONT: Chr1
d
ONT 300k: aneuploid
ONT 300k: aneuploid
ONT 300k: aneuploid
300000 reads
300000 reads
300000 reads
Read count
Read count
Read count
Chromosomes
Chromosomes
Chromosomes
e
ONT 300k: Chr1
ONT 300k: Chr2
ONT 300k: Chr7
