# Supplementary material for: Aneuploidy detection in pooled polar bodies using rapid nanopore sequencing
Source: J Assist Reprod Genet. 2024 Apr 20;41(5):1261–71. doi: 10.1007/s10815-024-03108-7 (PMC11143085; doi:10.1007/s10815-024-03108-7)
Supplement: Supplementary file 4 — Supplementary file4 (PPTX 960 KB) [file 10815_2024_3108_MOESM4_ESM.pptx]

## Slide 1
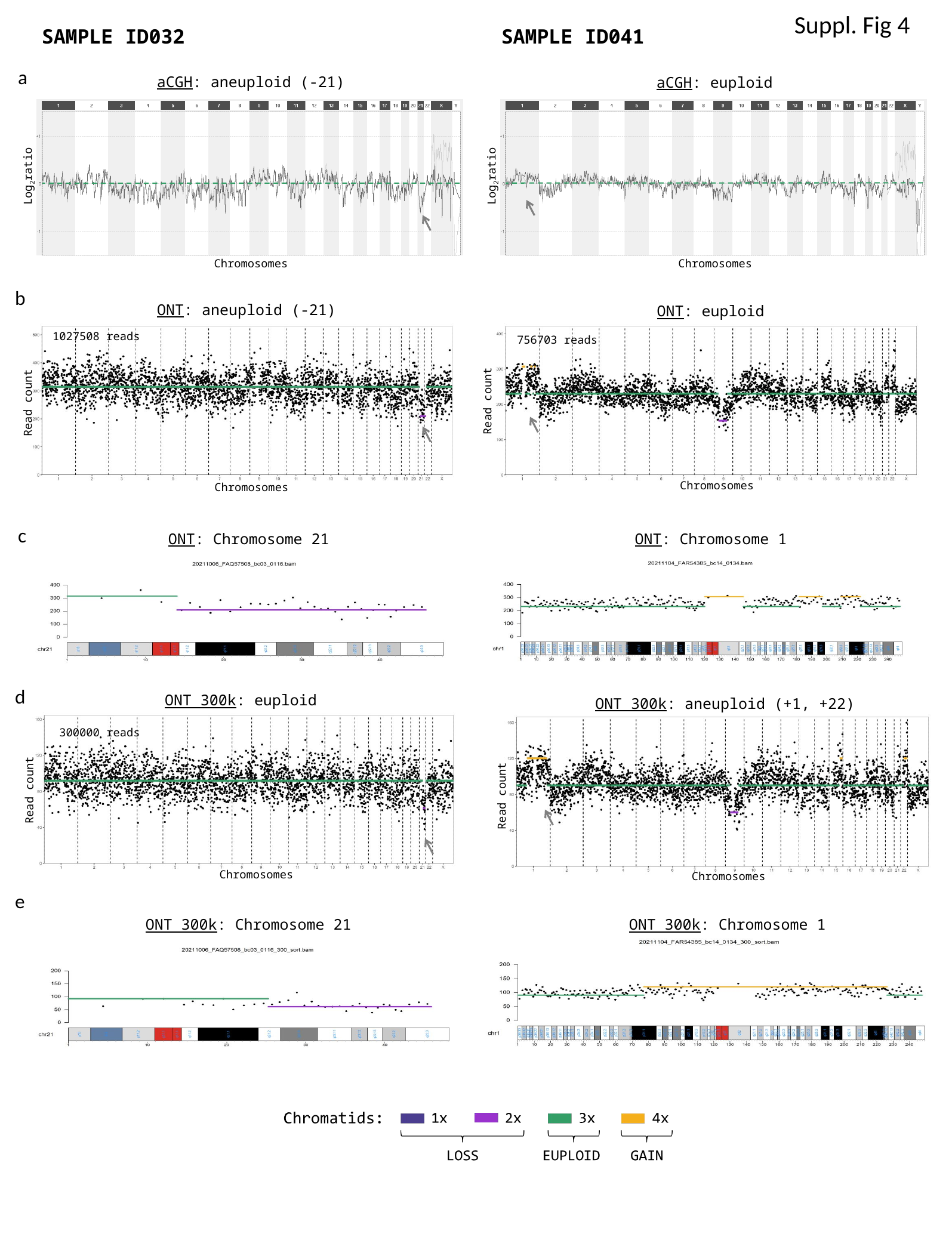

Suppl. Fig 4
SAMPLE ID032
SAMPLE ID041
a
aCGH: aneuploid (-21)
aCGH: euploid
Log2ratio
Log2ratio
Chromosomes
Chromosomes
b
ONT: aneuploid (-21)
ONT: euploid
1027508 reads
756703 reads
Read count
Read count
Chromosomes
Chromosomes
c
ONT: Chromosome 21
ONT: Chromosome 1
d
ONT 300k: euploid
ONT 300k: aneuploid (+1, +22)
300000 reads
300000 reads
Read count
Read count
Chromosomes
Chromosomes
e
ONT 300k: Chromosome 21
ONT 300k: Chromosome 1
