# Supplementary material for: Aneuploidy detection in pooled polar bodies using rapid nanopore sequencing
Source: J Assist Reprod Genet. 2024 Apr 20;41(5):1261–71. doi: 10.1007/s10815-024-03108-7 (PMC11143085; doi:10.1007/s10815-024-03108-7)
Supplement: Supplementary file 7 — Supplementary file7 (DOCX 15.0 KB) [file 10815_2024_3108_MOESM7_ESM.docx]

Supplementary information

**Supplementary Fig S1** Comparing ploidy analyses using aCGH and ONT for samples that were evaluated discrepantly. The ploidy classification based on the mean log_2_ ratio thresholds for aCGH and the reported clinical evaluation or the computed ploidy classifications with ONT are stated above.

a: Genome-wide analysis of the CNV state using aCGH.

b: Mean log_2_ ratios (sample vs. control DNA amplified by WGA) of each chromosome using aCGH. Light gray bars show signal distribution vs male control, dark grey bars that vs female control. Thresholds for a gain (orange line) and loss (blue line) for pooled polar bodies are indicated.

c: Genome-wide CNV analysis using ONT. Black dots represent the total read count per variable-width bin of approximately 1 Mb. A yellow line indicates a gain and a violet line indicates a loss or double loss, respectively.

d: Boxplot of binned read counts per chromosome using ONT. Coloured lines show the expected read count per state. The yellow line indicates the expected read count for a gain and the violet line indicates the expected read count for a loss, respectively.

**Supplementary Fig S2** Samples evaluated as highly complex by ONT

Genome-wide analysis of the CNV state using aCGH (left) and ONT (right) is shown. The blue dashed line represents the baseline (euploid state: 3 chromatids). In ID016, ID040, ID042, ID045, ID051, ID065, and ID071, a different baseline level (euploid state) can be observed, which shifts the number of assigned chromosomal gains in the ONT data. The pattern of the points measured by both methods is comparable, and all samples are aneuploid in both methods.

**Supplementary Fig S3** Genome-wide representation of the CNV states of selected samples showing segmental aneuploidy with a) aCGH, b) ONT, and a c) single chromosome representation of ONT as well as d) ONT and a e) single chromosome representation using the reduced data set (300 k reads). The sample ploidy classification based on the mean log_2_ ratio thresholds for aCGH or the computed ploidy classifications with ONT are stated above.

**Supplementary Fig S4** Genome-wide representation of the CNV states of selected samples with a) aCGH, b) ONT, and a c) single chromosome representation of ONT as well as d) ONT and a e) single chromosome representation using the reduced data set (300 k reads).

For a, b and d the ploidy classification based on the mean log_2_ ratio thresholds for aCGH, which are consistent with the reported clinical evaluation, or the computed ploidy classifications with ONT are stated above.

**Supplementary Table S1** Detailed information of the samples and obtained results

**Supplementary Table S2a** Number of chromosomes assigned to the specific ploidy classifications using the reduced data set (300 k reads) of ONT and aCGH in the form of a cross table (blue = concordant, red = discordant).

**Supplementary Table S2b** Number of chromosomes assigned to the specific ploidy classifications using the reduced data set (300 k reads) of ONT and the original ONT data (blue = concordant, red = discordant).
